# Establishment of a prognostic nomogram for elderly patients with limited-stage small cell lung cancer receiving radiotherapy

**DOI:** 10.1038/s41598-024-62533-x

**Published:** 2024-05-25

**Authors:** Lixia Zhang, Qingfen Zhang, Qian Wu, Lujun Zhao, Yunbin Gao, Xue Li, Song Guan, Meng Yan

**Affiliations:** Department of Radiation Oncology, Tianjin Medical University Cancer Institute & Hospital,National Clinical Research Center for Cancer, Tianjin’s Clinical Research Center for Cancer, Key Laboratory of Cancer Prevention and Therapy, Tianjin, 300060 China

**Keywords:** Elderly patients, Limited-stage small cell lung cancer, Nomogram, Survival prediction, Cancer, Oncology

## Abstract

The present study explored the risk factors associated with radiotherapy in seniors diagnosed with limited-stage small cell lung cancer (LS-SCLC) to construct and validate a prognostic nomogram. The study retrospectively included 137 elderly patients with LS-SCLC who previously received radiotherapy. Univariate and multivariate COX analyses were conducted to identify independent risk factors and determine optimal cut-off values. Kaplan–Meier survival curves and nomograms were constructed to predict survival. Calibration and receiver operating characteristic (ROC) curves were used to evaluate the accuracy and consistency of the nomogram. Illness rating scale-geriatric (CIRS-G) score, treatment strategy, lymphocyte-to-monocyte ratio (LMR), white blood cell-to-monocyte ratio (WMR), and prognostic nutritional index (PNI) were discovered to be independent prognostic factors. Based on the findings of our multivariate analysis, a risk nomogram was developed to assess patient prognosis. Internal bootstrap resampling was utilized to validate the model, and while the accuracy of the AUC curve at 1 year was modest at 0.657 (95% CI 0.458–0.856), good results were achieved in predicting 3- and 5 year survival with AUCs of 0.757 (95% CI 0.670–0.843) and 0.768 (95% CI 0.643–0.893), respectively. Calibration curves for 1-, 3-, and 5 year overall survival probabilities demonstrated good cocsistency between expected and actual outcomes. Patients with concurrent chemoradiotherapy, CIRS-G score > 5 points and low PNI, WMR and LMR correlated with poor prognosis. The nomogram model developed based on these factors demonstrated good predictive performance and provides a simple, accessible, and practical tool for clinicians to guide clinical decision-making and study design.

## Introduction

Lung cancer is one of the leading cause of cancer-related mortality^1^. Global cancer statistics^2,3^ for 2020 released by the International Agency for Research on cancer show that there were an estimated 820,000 newly diagnosed cases of lung cancer and 715,000 lung cancer-related deaths in China in 2020. The rise in life expectancy worldwide has resulted in a greater incidence of cancer cases among older adults. Based on available epidemiological data^4,5^ over 60–70% of individuals with lung cancer are aged 65 or older, and approximately 30–40% of new lung cancer cases are detected in patients aged 70 or above. About 15% of all lung cancer cases are attributed to small cell lung cancer (SCLC), one of the pathological subtypes^6^. SCLC is usually diagnosed in patients with a history of smoking, especially those over 65 years^7^^.^

Despite significant advancements in the management of non-small cell lung cancer (NSCLC) and that of extensive small cell lung cancer (ES-SCLC), the approach to treating LS-SCLC has seen limited progress in recent years. Except that only 5% patients are eligible for surgery, the most common treatment for LS-SCLC is still the combination of cisplatin and etoposide (EP regimen) with concurrent chemoradiotherapy (CCRT)^8^. However, elderly patients are often deprived of standard care due to various factors, resulting in a poorer prognosis.

Age is considered an independent prognostic factor for SCLC patients^9,10^, as elderly patients often experience complications, organ function aging, physical and nutritional decline, and reduced immunity^11^. In clinical practice, the evaluation of complications in elderly patients often involves commonly used assessment tools such as the Charlson comorbidity index (CCI), age-adjusted Charlson comorbidity index (A-CCI), and cumulative illness rating scale-geriatric (CIRS-G)^12^. While these indicators have been linked to the prognosis of elderly patients, some scores are subjective and require the addition of objective clinical indicators for a comprehensive prognosis. Prognostic nutritional index (PNI) has been used to measure both nutritional and immune status in several studies^13^. It has shown a correlation with the prognosis of advanced NSCLC^14^, esophageal cancer^13^, and gastric cancer^15^. However, limited research has been conducted on PNI in elderly LS-SCLC patients. Studies have shown that lung cancer development is closely linked to the inflammatory microenvironment of tumors, and inflammatory cells and factors can promote tumor progression, metastasis, and immune evasion through the activation of inflammatory signaling pathways^16^. Currently, hematologic and biochemical markers reflecting systemic inflammatory response are easily accessible and can be measured in routine blood tests^17^. Therefore, they are being increasingly recognized for their clinical value in cancer patients. Several studies have demonstrated that these markers, including neutrophil-to-lymphocyte ratio (NLR), platelets-to-lymphocyte ratio (PLR) and lymphocyte-to-monocyte ratio (LMR), are significantly correlated with prognosis in patients with resectable lung cancer^18^, locally advanced colorectal cancer^19^, non-muscular invasive bladder cancer^20^ and other cancers. Moreover, other inflammatory markers that combine WBC, such as WLR, WHR, WNR, WMR, and WRR, have also been investigated to establish their association with bladder cancer prognosis^21^. However, there is a paucity of research on using these inflammatory markers in senior LS-SCLC patients.

In summary, there is a significant clinical need to investigate accessible and objective prognostic factors for elderly LS-SCLC patients. Nomograms are considered reliable tools for assessing prognosis and risk by integrating important clinical features. However, currently available models were not specifically developed for the elderly population. Therefore, constructing a nomogram specifically for elderly patients with LS-SCLC is necessary to aid in this cohort’s survival prediction.

## Materials and methods

### Study population

From April 2011 to August 2020, a total of 137 elderly patients with LS-SCLC who underwent treatment at the Cancer Institute and Hospital of Tianjin Medical University Cancer Institute & Hospital Medical University were enrolled in this study according to the inclusion criteria. The inclusion criteria for this study were as follows: (1) patients ≥ 65 years of age; (2) pathologically confirmed SCLC; (3) patients were classified as LS-SCLC according to the Veterans Administration Lung Study Group Reassessment (VALG) staging criteria; (4) patients who received concurrent or sequential chemoradiotherapy (SCRT); (5) patients with complete history information, imaging and laboratory tests (routine blood, liver and kidney function tests). Patients with hematologic disease and active infection were excluded. Before treatment, all patients underwent chest CT scan and contrast-enhanced MRI, and some patients suspected of metastasis underwent PET-CT scan.This study was carried out after obtaining approval from the Tianjin Medical University Cancer Institute & Hospital ethics committee with ethics approval number bc2023076.

### Treatment stategy

All patients included in this study received multidisciplinary consultation before starting treatment. For patients who are in good physical condition after evaluation, we adopted the strategy of CCRT. For patients with poor physical condition who cannot tolerate SCRT was adopted. Induction chemotherapy was given to patients with large tumor volume or suspected lymph node metastasis. The induction chemotherapy regimen was: etoposide combined with cisplatin or carboplatin, 2 cycles. The radiotherapy method was: intensity-modulated radiotherapy (IMRT), 60 Gy/30f.

### Study variables

The study assessed several variables, including age, sex, smoking history, weight loss, KPS score, A-CCI score, CIRS-G score, T stage,N stage, radiotherapy regimen including CCRT, SCRT, induction chemotherapy and prophylactic cranial irradiation (PCI), hematological indicators such as routine blood tests and albumin at the start of treatment, duration of follow-up, and all-cause mortality. Patients with incomplete information on the aforementioned variables were excluded from the study. Overall survival referred to the period between the time from diagnosis to last follow-up or death.

The included serum indicators were as follows: neutrophil-to-lymphocyte ratio (NLR), platelets-to-lymphocyte ratio (PLR), lymphocyte-to-monocyte ratio (LMR),white blood cell-to-lymphocyte ratio (WLR), white blood cell-to-hemoglobin ratio (WHR), white blood cell-to-neutrophil ratio (WNR), white blood cell-to-monocyte ratio (WMR), white blood cell-to-erythrocyte ratio (WRR) and prognostic nutritional index (PNI). Notably, PNI was quantified using 10 × serum albumin (g/dL) + 0.005 × total lymphocytes count (/mm^3^). The absolute counts of each parameter were used to calculate all of the ratios.

### Statistical analysis and nomograph construction

Frequency and percentage were utilized for categorical variables. In contrast, median with interquartile range (IQR) was utilized for continuous variables. Univariate and multivariate Cox two-way stepwise analyses were conducted to determine if the correlated variables were independent predictors of overall survival (OS). The study determined the best cut-off values for the detected independent factors using the surv_cutpoint function in R with OS as the endpoint indicator. The variables were grouped according to the cut-off values, and survival curves were evaluated using the Kaplan–Meier method, which were then compared using the log-rank test. And p-value below 0.05 was deemed to be significant.

Additionally, a nomogram was constructed using the independent prognostic factors detected in the multivariate analysis, and bootstrap resampling was performed for internal validation. The nomogram’s predictive efficacy was evaluated using the AUROC, and a calibration curve was drawn to determine the agreement between the actual results and the predicted survival rate for elderly patients with limited-stage small-cell lung cancer.

The statistical analysis for all data was performed using the R statistics program (R Core Team, 2022), an environment and language for statistical computing. The program is available at https://www.R-project.org.

### Statement

All methods were carried out in accordance with relevant guidelines and regulations.

### Consent to participate

The study confirmed that informed consent was obtained from all subjects and/or their legal guardians.

## Results

### Patients characteristics

The study included 137 eligible patients based on predetermined inclusion and exclusion criteria. Among them, 96 (70.1%) were male, with a median age of 68 (65–84). CCRT was administered to 71 (51.8%) patients, while SCRT was given to 66 (48.2%) patients. Induction chemotherapy was not given to 12 patients, and only 10 patients underwent prophylactic cranial irradiation (PCI). Patient characteristics are detailed in Table1.

**Table 1:** Baseline characteristics of the patients.

Characteristic	N = 137
Age (years)	
Median	68
Range	65–84
Sex	
Male	96 (70.1%)
Female	41 (29.9%)
Smoking history	
Yes	101 (73.7%)
No	36 (26.3%)
Weight loss	
Yes	27 (19.7%)
No	110 (80.3%)
KPS score	
≤ 80	70 (51.1%)
> 80	67 (48.9%)
A-CCI score	
2	69 (50.4%)
3	51 (37.2%)
4	16 (11.7%)
5	1 (0.73%)
CIRS-G score	3.00 [2.00; 5.00]
T stage	
T1	18 (13.1%)
T2	74 (54.0%)
T3	27 (19.7%)
T4	18 (13.1%)
N stage	
N0	4 (2.9%)
N1	11 (8.0%)
N2	94 (68.6%)
N3	28 (20.4%)
Radiotherapy regimen	
CCRT	71 (51.8%)
SCRT	66 (48.2%)
Induction chemotherapy	
Yes	125 (91.24%)
No	12 (8.76%)
PCI	
Yes	10 (7.5%)
No	124 (92.5%)
NLR	2.34 [1.65; 3.35]
PLR	139.39 [107.10; 180.00]
LMR	3.28 [2.29; 4.37]
WLR	3.78 [2.97; 4.78]
WHR	0.05 [0.04; 0.06]
WNR	1.60 [1.45; 1.84]
WMR	12.05 [10.04; 14.93]
WRR	1.45 [1.17; 1.77]
PNI	49.90 [46.90; 54.60]

*KPS* Karnofsky performance status, *CCI* Charlson comorbidity index, *A-CCI* age-adjusted charlson comorbidity index, *CIRS-G* illness rating scale-geriatric, *CCRT* concurrent chemoradiotherapy, *SCRT* sequential chemoradiotherapy, *PCI* prophylactic cranial irradiation.

### Independent prognostic factors

**Figure 1:**
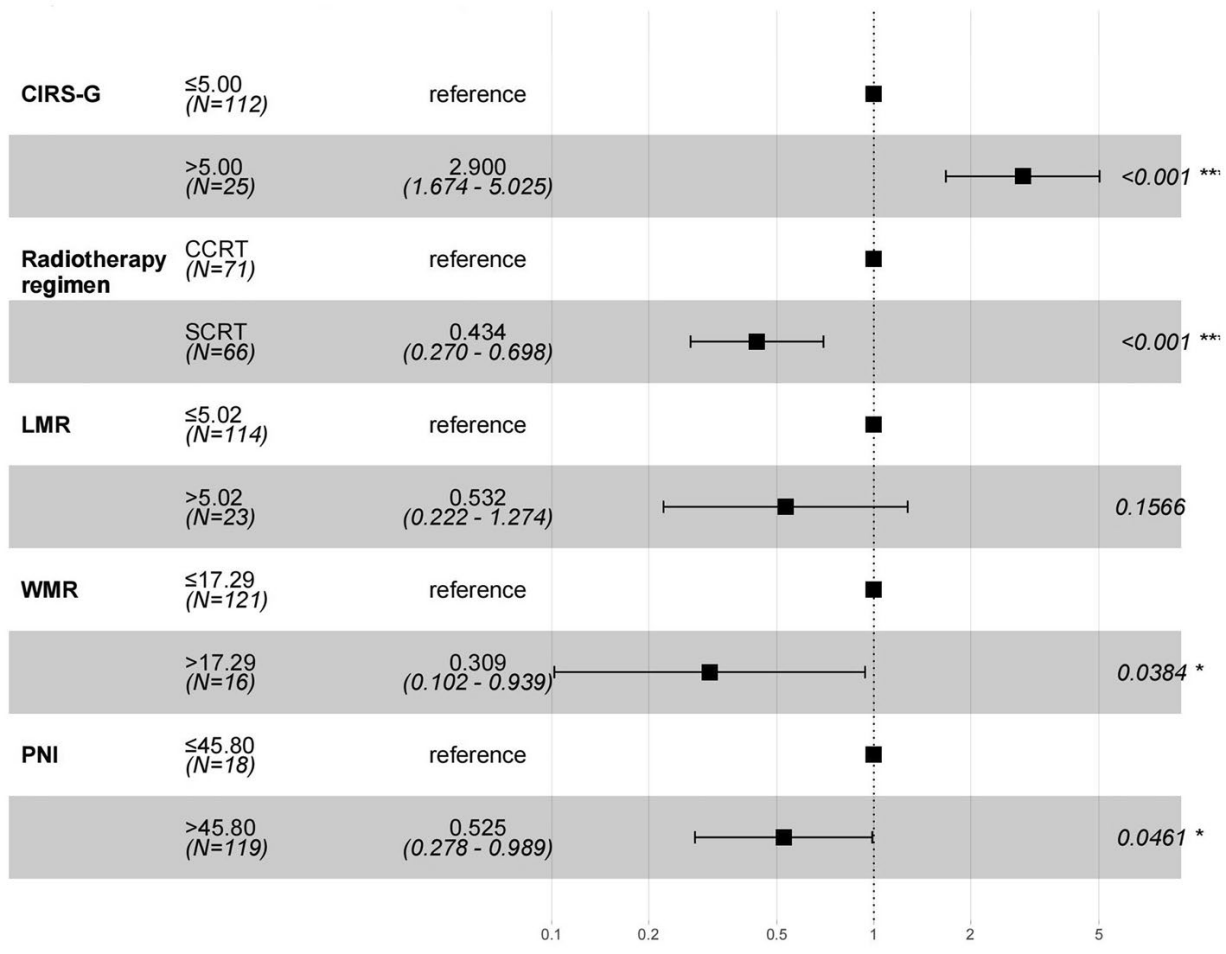
Multivariate Cox regression analysis model for overall survival.

Table 2 presents the findings of our univariate analysis, and Fig. 1 displays the findings of the multivariate analysis. In the univariate analysis, gender (p = 0.031, HR = 0.558), CIRS-G score (p = 0.001, HR = 2.460), radiotherapy regimen (p = 0.038, HR = 0.620), NLR (p = 0.016, HR = 1.767), LMR (p = 0.003, HR = 0.328), WLR (p = 0.019, HR = 1.744), WMR (p = 0.007, HR = 0.281), and PNI (p = 0.028, HR = 0.497) were significant, progressing to our multivariate analysis. Further analysis revealed that CIRS-G score (p < 0.001, HR = 2.900), radiotherapy regimen (p < 0.001, HR = 0.434), LMR (p = 0.157, HR = 0.532), WMR (p = 0.038, HR = 0.309), and PNI (p = 0.046, HR = 0.525) were independent prognostic factors for overall survival (Fig. 1).

**Table 2:** Univariate Cox regression analysis model for overall survival.

Variables	HR (95% CI)	P-value
Age (years)	1.018 (0.958, 1.082)	0.558
Sex		
Male	–	–
Female	0.558 (0.328, 0.947)	0.031
Smoking history		
No	–	–
Yes	1.494 (0.879, 2.539)	0.138
Weight loss		
No	–	–
Yes	1.514 (0.901, 2.543)	0.117
KPS score		
≤ 80	–	–
> 80	1.173 (0.752, 1.830)	0.481
A-CCI score		
2	–	–
3	1.301 (0.815, 2.075)	0.270
4	1.038 (0.463, 2.328)	0.927
5	0.000 (0.000, Inf)	0.997
CIRS-G score		
≤ 5.00	–	–
> 5.00	2.460 (1.455, 4.159)	0.001
T stage		
T1	–	–
T2	1.216 (0.591, 2.501)	0.595
T3	1.090 (0.476, 2.494)	0.839
T4	1.637 (0.675, 3.970)	0.276
N stage		
N0	–	–
N1	0.274 (0.039, 1.950)	0.196
N2	1.553 (0.378, 6.371)	0.541
N3	1.438 (0.333, 6.207)	0.626
Radiotherapy regimen		
CCRT	–	–
SCRT	0.620 (0.394, 0.975)	0.038
Induction chemotherapy		
No	–	–
Yes	0.868 (0.417, 1.808)	0.705
PCI		
No	–	–
Yes	0.617 (0.248, 1.536)	0.300
NLR		
≤ 2.06	–	–
> 2.06	1.767 (1.114, 2.802)	0.016
PLR		
≤ 89.52	–	–
> 89.52	1.369 (0.658, 2.848)	0.401
LMR		
≤ 5.02	–	–
> 5.02	0.328 (0.157, 0.686)	0.003
WLR		
≤ 3.33	–	–
> 3.33	1.744 (1.098, 2.772)	0.019
WHR		
≤ 0.03	–	–
> 0.03	1.747 (0.757, 4.035)	0.191
WNR		
≤ 1.58	–	–
> 1.58	0.643 (0.411, 1.006)	0.053
WMR		
≤ 17.29	–	–
> 17.29	0.281 (0.112, 0.706)	0.007
WRR		
≤ 1.16	–	–
> 1.16	1.531 (0.880, 2.665)	0.132
PNI		
≤ 45.80	–	–
> 45.80	0.497 (0.267, 0.927)	0.028

### Survival analysis

**Figure 2:**
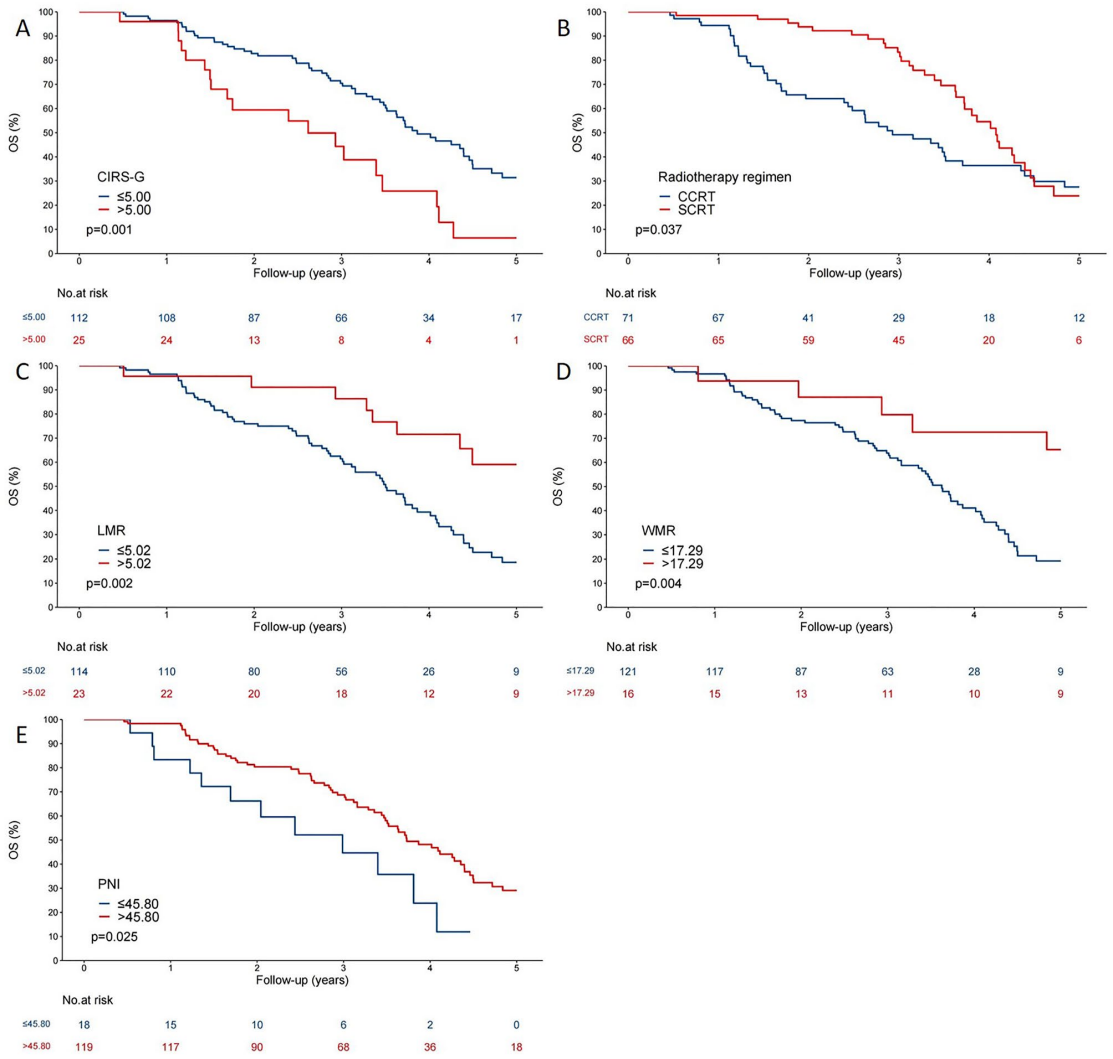
The KM curves of inflammatory marks for elderly patients with limitated-stage small cell lung cancer. (**A**–**E**) KM curve was made to exhibit the prognosis of the different therapy regimen, CIRS-G score, and diferent expression level of LMR, WMR, and PNl, respectively.

Patients were followed up for a median of 3.16 years. Optimal cut-off values for each independent prognostic factor were determined using the surv_cutpoint function, and Kaplan–Meier (KM) curves were generated to illustrate the prognostic outcomes of different CIRS-G, radiotherapy regimen, LMR, WMR, and PNI (Fig. 2A–E). The results indicate that patients receiving CCRT, those with CIRS-G scores greater than 5, and those with low PNI, WMR, and LMR values had a poorer prognosis in elderly LS-SCLC patients.

### Nomogram construction

**Figure 3:**
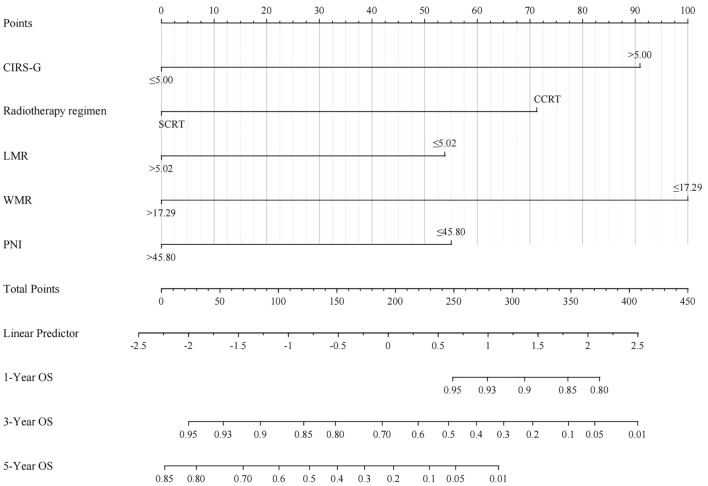
Nomograms to predict 3- and 5 year overall survival. A vertical line can be drawn belween each varable and the points scale toacauire the points of each varable. Predicted survival rate was calculated according to the total points by drawing a vertical ine from the total points scale to the overall survival scale.

The study developed a predictive model for elderly LS-SCLC patients based on the five independent risk factors identified earlier. The model includes 1-, 3-, and 5 year predictions and is depicted in Fig. 3. The total score was calculated by adding up the scores of each predictor variable, and the corresponding survival rate can be estimated by finding the intersection of the total score with the vertical line of the predicted survival rate on the graph.

### Calibration and internal validation

**Figure 4:**
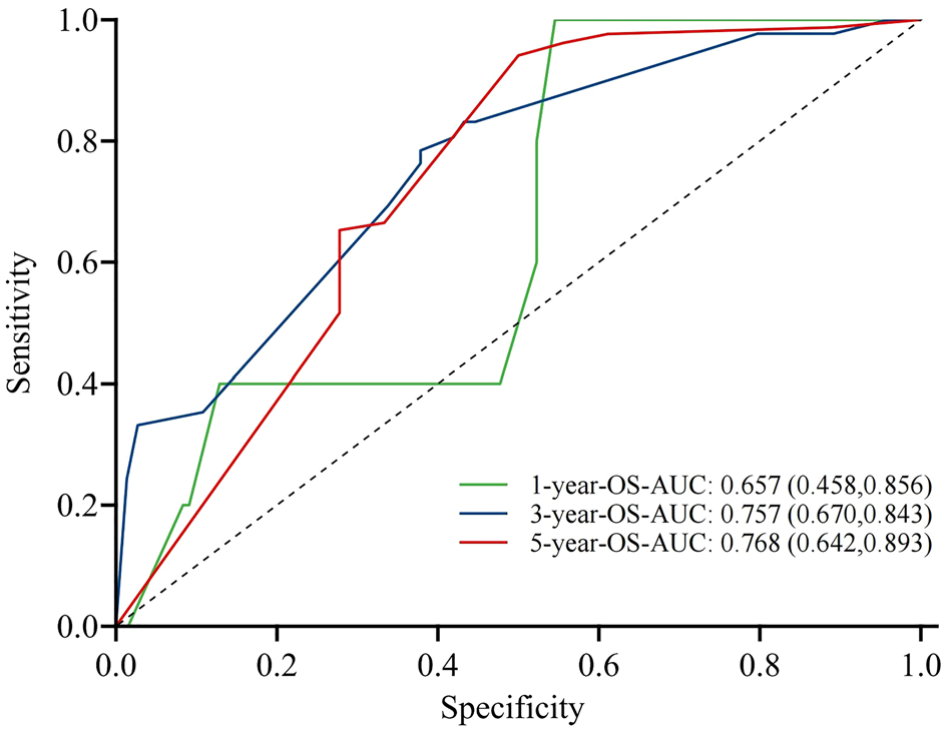
ROC curve of prognostic model of elderly LS-SCLC.

**Figure 5:**
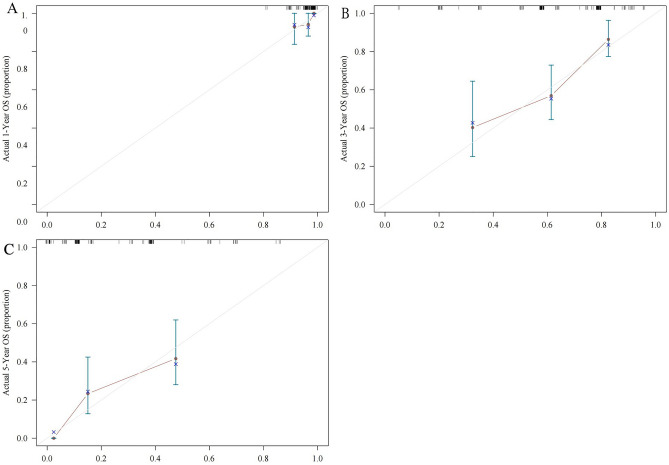
Nomogram-predicted probability of 1 year (**A**), 3 year (**B**), and 5 year (**C**) overall survival.

The nomogram exhibited superior predictive performance, as demonstrated by the 1-, 3-, and 5 year ROC curves for OS generated using the score function in the risk regression package. The areas under the ROC curve (AUROC) were 0.657 (95% CI 0.458, 0.856), 0.757 (95% CI 0.670, 0.843), and 0.768 (95% CI 0.642, 0.893) (Fig. 4). Furthermore, accuracy of the prediction model was assessed by calibration curves, which indicated good agreement between the predicted and actual survival rates (Fig. 5A–C). These results collectively suggest that the nomogram developed in this study can be effectively used to evaluate the prognosis of elderly LS-SCLC patients.

## Discussion

The National Comprehensive Cancer Network, NCCN guidelines define elderly patients as those aged 65 or older^22^. Given the heterogeneity of this population, the treatment and prognosis of limited-stage small cell lung cancer in elderly patients pose a significant challenge. Therefore, conducting a thorough prognostic analysis for this group is valuable. Conversely, there is a limited amount of research on prognostic models for elderly LS-SCLC patients.

The findings of this study suggest that the CIRS-G score, type of therapy used, LMR, WMR, and PNI are important prognostic factors for elderly patients with limited-stage small-cell lung cancer. In clinical practice, the assessment of geriatric comorbidity is typically achieved using scales such as the CCI, A-CCI, and CIRS-G^12^. The A-CCI score incorporates age-related factors into the CCI score and is more appropriate for studying comorbidities in elderly patients. Meanwhile, the CIRS-G score, which evaluates 14 organ systems, is the recommended scale according to the comprehensive geriatric assessment (CGA). The A-CCI score is derived from the CCI and increases by one point every 10 years after the age of 40^23^. Meanwhile, the CIRS-G is a cumulative rating score that assesses 14 organ systems, with a severity rating ranging from 0 (no problem) to 4 (very serious) for each system^12^. Herein, the assessment of geriatric comorbidity utilized both A-CCI and CIRS-G.Our analysis revealed that a CIRS-G score > 5 was synonymous of a poor prognosis for our LS-SCLC patients. Recent studies have also found that elderly patients with mantle cell lymphoma (MCL) and a high CIRS-G score (p = 0.04, HR = 1.06) have worse overall survival^24^. Concurrent radiotherapy is theoretically as effective in elderly patients with LS-SCLC, but treatment tolerability is an important consideration. Research has shown that standard treatment in older patients increases the risk of toxicity, requiring a comprehensive evaluation and timely intervention^25^. Although many studies have shown that patients receiving CCRT have a high survival rate with SCRT^26–28^, this survival benefit is usually at the expense of greater therapeutic toxicity. Many studies have confirmed that SCRT is less toxic to patients’ organs and hematologic system than CCRT^29,30^. Even in some studies, the survival rate of patients receiving SCRT was significantly better than that of patients receiving CCRT^31,32^. Similarly, the results of this study showed that the survival rate of patients receiving SCRT was significantly higher than that of patients receiving CCRT, which may be due to the strong therapeutic toxicity caused by CCRT.

LMR and WMR, as systemic inflammatory response markers are often linked with cancer prognosis, with low levels of LMR and WMR expression correlating with a poor outcome for patients with bladder cancer (BLCA)^21^, which is in line with the findings of our study. The PNI is an index used to assess nutritional status, which is based on both albumin levels and lymphocyte counts. It serves as a biological marker indicative of the nutritional status of the body and the degree of the inflammatory response^13^. Park et al.^33^ showed that the lower the PNI value, the greater the risk of postoperative delirium in patients undergoing radical lung cancer surgery. It has also been shown that PNI is related to clinical outcomes in hepatocellular carcinoma, with a decrease in PNI associated with a worse OS (HR = 0.31, 95% CI 0.17–0.57; P = 0.02)^34^. Consistently, our study in elderly patients with small cell lung cancer also found that PNI is significantly associated with prognosis. Notably, a decrease in PNI value indicates malnutrition and increased inflammatory response, which is associated with poor prognosis.

To conclude, this study presents a straightforward and all-encompassing prognostic model for elderly patients with limited-stage SCLC using fundamental clinical characteristics. Unlike most existing models, this nomogram was specifically designed for a group of elderly LS-SCLC patients, and all indicators used are economical and readily accessible.This provides a predictive model that accurately represents the survival of elderly LS-SCLC patients, thereby guiding clinicians in treatment selection and providing evidence for further clinical studies.

Nevertheless, our report has some limitations. First, the sample size for our training cohort was quite small; thus, it is necessary to expand the cohort size in future studies. Secondly, there is a lack of data for external verification. While our study suggests caution in using CCRT in elderly patients, further prospective data are needed to assess psychological, social, and other factors using comprehensive geriatric assessment (CGA) when deciding which older adults get standard treatment.

## Conclusion

To summarize, there is currently a lack of prognostic models for elderly LS-SCLC patients. The nomogram developed in this study can provide a preliminary understanding of the prognosis of this patient population. However, further validation through prospective studies and external validation is necessary to substantiate our nomogram’s robustness.

## Data Availability

The data that support the findings of this study are available from Tianjin Medical University Cancer Institute & Hospital but restrictions apply to the availability of these data, which were used under license for the current study, and so are not publicly available.If someone wants to request for the raw data used in this study will be fulfilled from the corresponding author (Dr. Lujun Zhao).
